# Supplementary material for: Are carbon emissions trading and green financial instruments synergistic? -Comprehensive quantitative research based on content analysis
Source: PLoS One. 2024 Mar 7;19(3):e0298601. doi: 10.1371/journal.pone.0298601 (PMC10919601; doi:10.1371/journal.pone.0298601)
Supplement: S1 Data — (ZIP) [file pone.0298601.s001.zip › Data/地级市Z_F工作报告.zip!/╣π╢1⁄2/╓╨╔╜╩╨/└┤╫╘╣1⁄2╓┌║┼í╛┬φ┐╦╩2╛▌═°í┐_2007.docx]

各位代表：

　　我代表市人民政府向大会作政府工作报告，请予审议，并请政协委员和其他列席人员提出意见。

　　过去三年工作回顾

　　本届政府任期三年来，在省委、省政府和市委的正确领导下，高举邓小平理论和“三个代表”重要思想伟大旗帜，深入贯彻落实党的十六届四中、五中、六中全会和胡锦涛总书记、温家宝总理以及张德江书记视察中山的重要讲话精神，坚持科学发展观，大力推进和谐社会建设，坚持“工业强市”、“依法治市”、“科教兴市”和可持续发展战略，迎难而上，保持了全市经济社会快速健康发展态势，“十五”计划确定的各项任务全面完成，市十二届人大一次会议确定的预期目标已经实现，建设全省经济社会协调发展示范市取得阶段性成果，建设“两个适宜”城市、构建和谐中山初显成效。

　　综合经济实力跃上新台阶，生产总值突破千亿元。2006年全市生产总值达1034亿元（预计，下同），年均增长20.2%，户籍人均生产总值 73100多元，三次产业比例调整为3.1：61.1:35.8。2006年地方财政一般预算收入、社会消费品零售总额分别达66.4亿和329.1亿元，比2003年增长81.5%和63.4%。工业保持较快增长，质量效益同步提高，2006年全市工业总产值超3000亿元，年均增长23.2%，规模以上工业企业经济效益综合指数连续三年超过130%。产业高级化、适度重型化趋势更加明显，高新技术产品产值和装备制造业产值分别比2003年增长 174.8%和108.1%，2006年重工业在轻重工业总量所占的比例比2003年提高7个百分点。产业集群优势进一步增强，拥有22个国家级产业基地、11个省级专业镇、7个广东省产业集群升级示范区。三年来共安排2.3亿元用于技改贴息、扶持装备制造业、中小企业发展和推进名牌战略，其中投入技改贴息补助资金1.04亿元，带动投资79.4亿元，企业创新能力和竞争力得到提高。名牌带动战略成效显著，累计获得国家、省名牌名标、免检产品344个，被评为“中国品牌经济城市”和“全国质量兴市先进市”。资源配置得到优化，与阳江、韶关、河源等市合作建设规划总面积达3.8万亩的4个产业转移园区。切实提高土地集约利用水平，大力发展循环经济，综合能耗水平不断下降。服务业加速发展，2006年服务业增加值比2003年增长74.7%，服务业占生产总值比重持续提高。农村工业化、城镇化和农业产业化步伐加快，综合生产能力增强，效益农业、特色农业、品牌农业和绿色农业发展态势良好，农业科技贡献率达 60%，共有市级以上农业龙头企业27家，2006年实现农业总产值54亿元，比2003年增长13.4%。

　　外经贸增长方式有新变化，内外源经济协调快速增长。紧紧抓住全球产业转移和泛珠合作的机遇，扩大对外开放，利用外资结构不断优化。累计批准外商直接投资项目1066宗，实际利用外资18.7亿美元，其中投资超千万美元项目54宗。坚持扩大外贸出口和进口相结合，累计实现外贸进出口575亿美元。坚持完善进出口公平贸易工作机制，反倾销预警和贸易壁垒应对工作取得成效。口岸设施不断完善，通关效率进一步提高。“走出去”步伐加快。民营经济迅速发展，全市共有民营企业1.8万家，个体工商户11.2万户，2006年民营企业实现工业总产值1063.8亿元，占全市工业总产值的35.5%。

　　基础设施建设取得新进展，城市功能更加完善。按照城乡一体、彰显特色、拉大框架、完善功能的思路，投入18亿元建成一批城市标志性工程，城市化率达 74.3%。交通方面，加快建设干线公路网，配合省建设中江高速、西部沿海高速等公路，南外环城南路以东段、城桂公路二期等重点项目完工。坚持公交优先战略，公交站场、线路建设成效明显，完成博爱路和起湾道等城区道路改造。“畅通工程”、“智能交通系统工程”通过验收，全市公路通车总里程1665.8公里，港口集装箱吞吐量达113万标准箱，中山港继续位列全国港口集装箱吞吐量前10强。能源方面，完成香山500千伏等一批输变电工程，南朗、永安电厂和天然气管道主工程相继建成，三年间全市供电总量达353.7亿千瓦时。信息化方面，全市光纤总皮长1.3万多公里，互联网用户59.9万户，信息化指数达 78%。环保方面，中心组团垃圾综合处理基地、中嘉污水处理厂二期等工程基本建成，新增垃圾和污水处理能力1100吨/日和15万吨/日。水利方面，投入 11.7亿元建设276宗城乡水利防灾减灾工程，成功抗击2005年百年一遇特大洪水。民生方面，建成文化艺术中心、市职业技术学院二期、中心粮库一期、疾控中心等工程，博览中心、新中医院等一批重点工程相继启动。完成16个“城中村”共1.2万户的供水管网改造，长江水厂建成并投入使用。农村基础设施方面，市级投入1.3亿元推进“农村五项工程”，新建4米宽以上农路703.4公里，农桥335座，涵闸142座，整治低产鱼塘20万亩，整治内河涌593 公里，完成“一区三线”林相改造4万亩，“四旁”绿化1735公里。东部开发、组团发展方面，中山港二期扩建工程完成可行性研究和港区总体规划编制，临海工业园配套基础设施基本建成，保税物流中心等重大项目加快推进，东部“一区三镇”生产总值占全市经济总量的23.1%。

　　各项改革取得新突破，体制创新活力增强。深化行政管理体制改革，设立五桂山街道办事处，推进火炬区与南朗镇资源整合。国有资产监督管理体制进一步完善，公有企业累计清减债务57.5亿元。加快构建公共财政框架，部门预算、国库集中支付、政府采购及绩效评价等工作全面推进。积极增加财政收入，三年来共实现市级非税收入95.1亿元。积极发展和利用资本市场，中小金融机构正加大力度退出市场，农信社改革取得成效，全市共有3家公司在国内上市。推进投融资体制改革，探索城市公共设施代建制等投融资建设模式。积极开展城市经营，采石场经营、出租车运营、公路广告经营等公共资源通过招投标或拍卖等方式进入市场。全面实施农村“三个一”工程，继续深化农村税费改革，全面免征农业税，实现农民农业税赋零负担。

农村股份合作制改革取得重大突破，全市有95.4%的村（居）、组建立了农村股份合作制。

　　关注民生有新进步，富裕安康工程全面实施。统筹城乡就业，全市共投入6000多万元用于培训、促进就业和再就业，转移农村富余劳动力实现就业5.9万人，城镇登记失业率控制在2.5%以下。全市职工最低月工资标准提高到690元，比2003年增长53.3%。社保覆盖面进一步扩大，社会保险参保达 371.2万人次，比2003年增长37.4%。大力推进农村基本养老保险和城乡居民住院基本医疗保险，至2006年底参保人数分别达34.9万人和19 万人，新型农村合作医疗覆盖率达97.1%。实施城乡孤寡老人集中供养工程，投资6300多万元改造扩建11家镇级农村敬老院，集中供养率达41%。推进扶危助困工程，设立“扶困助学基金”，累计建设“双困户”住房2.4万平方米，改造低保危房3.9万平方米。为促进镇区协调发展，三年间财政共转移支付 3.2亿元，向镇区提供各类贷款贴息资金1.1亿元。加大对经济欠发达村扶持力度，12个集体经济欠发达村实现经济纯收入达50万元以上。城乡居民生活水平明显提高，2006年城乡居民储蓄存款余额达832.8亿元，城镇居民年人均可支配收入18630元，农民人均年纯收入9006元，分别比2003年增长53.2%、25%和38.1%。

　　社会事业发展取得新成就，文明法治水平明显提高。坚持“科教兴市”战略，大力推动城市创新体系建设，市级投入科技发展经费3.17亿元，建成国家、省、市级工程技术研究开发中心121家，博士后工作站3个，火炬区创业中心被授予“国家级创业服务中心”称号。成功创建省“教育强市”，全市教育经费投入共67.7亿元，薄弱学校改造任务全面完成，中小学优质学位覆盖率近80%，高中和高等教育毛入学率达75.3%和43.8%，对全市户籍学生实施免费义务教育。香山文化研究和应用取得丰硕成果，公共文化服务体系日臻完善，镇区文化站全部达到省一级以上标准，一批博物馆建成开馆，成立中山日报报业集团，成功举办博鳌亚洲论坛2005文化产业会议。体育事业蓬勃发展，获得“全国全民健身样板城市”称号，获得团体及个人世界冠军5个、全国冠军25个。积极创建全国生态市和广东省林业生态市，设立五桂山自然生态保护区，推进“绿化中山大地工程”，大力开展清洁生产和治污保洁，建成“国家级生态示范区”，通过“国家环保模范城市”复检。继续完善医疗卫生服务体系、公共卫生体系、医疗急救体系，市镇村三级公共卫生服务网络进一步完善，共有国家、省级卫生镇18个，社区卫生服务面达81%。建立完善动物疫病防控体系，成功防控禽流感等疫情。加大城市管理力度，全面开展清理违法建筑和查处取缔无证照经营活动。扎实推进现代公民教育，弘扬“博爱、创新、包容、和谐”的新时期中山人精神，成功创建“全国文明城市”。大力推进“平安中山”建设，“六好”平安和谐社区共71个，信访网络进一步完善，社会矛盾有效缓解。抓好食品、药品、交通、消防等安全管理，安全生产形势良好。健全社会治安防控体系，初步建立起市、镇、村（居）三级流动人口管理体系。启动建设政法网（金盾工程）、社会治安视频监控系统，获得全国治安综合治理优秀地市“长安杯”称号，社会大局稳定。全市常住人口出生率为9.88‰，计划生育率为93.67%。

　　政府自身建设有新气象，依法行政和政务公开得到加强。深入贯彻落实《行政许可法》和《全面推进依法行政实施纲要》，制定和颁布了一批市政府规范性文件，设立政府法律顾问室。自觉接受市人大及其常委会的法律监督和市政协的民主监督，坚持重大事项向市委、市人大报告制度，建立市政府领导牵头办理重点议案、提案制度。完善“人大代表建议和政协提案网络办理在线系统”，共办理人大代表议案、建议395件、政协委员提案490件，答复率100%。推进新一轮审批制度改革，精简审批事项963项，35个部门的210个审批事项实现网上办理。继续推进政务公开，《政报》、“政府之窗”网站等平台不断完善，整合开通“12319”行政服务咨询热线，城市论坛成为了解民意、汲取民智、调动广大市民参政议政的新亮点。成立市公共突发事件应急联动委员会，不断提高政府应急管理水平。健全公开征求意见、专家咨询论证和决策听证等制度，就污水处理费征收等多项公共事务进行了听证工作。坚持市长接待日制度，处理群众来信来访事项1.1万宗，办结率达95%以上。加强公务员队伍和机关自身建设，行政效率和服务水平有新提高。深入推进先进性教育和排头兵实践活动，建设为民、务实、勤政的公务员队伍。大力推进廉政制度建设，基本完成公车改革。

　　此外，外事、侨务、台务、审计、统计、老干、气象、国防、档案、地方志、妇女儿童、残疾人、民族宗教、老区建设等各项工作也取得了新的成绩。

　　各位代表，过去三年，是我市经济快速健康发展的三年，是城乡建设和环境建设取得巨大进步的三年，是社会管理和文化建设取得全面进步的三年，是人民群众得到较多实惠、民生明显改善的三年。回顾三年来的工作，我们有以下体会：一是必须始终坚持以科学发展观统揽全局，着力推动经济和社会的理性发展、协调发展；二是必须始终坚持通过加快发展不断破解前进中的难题，以体制机制创新增强发展后劲，着力解决好事关全局的长远性和战略性问题；三是必须始终坚持走自己的路，着力在激烈的竞争中彰显“中山特色”和比较优势；四是必须始终坚持执政为民，把维护人民群众的利益作为各项工作的出发点和落脚点，着力解决好人民群众最关心、最直接、最现实的利益问题；五是必须始终坚持依法行政，把加强政府自身建设作为提供良好公共服务的组织保障，着力提升城市文明程度，促进经济社会事业全面进步。

　　各位代表，历届市委、市政府团结带领全市人民，用辛劳和智慧、务实和拼搏，谱写了中山发展史上一篇又一篇精彩华章。三年来取得的工作成绩来之不易，这是省委、省政府和市委正确领导的结果，是市人大及其常委会依法监督，市政协以及各民主党派民主监督和支持的结果，是全市人民群众努力奋斗的结果。我谨代表市政府向全市广大工人、农民、知识分子、干部职工、驻我市人民解放军、武警官兵、人民警察和社会各界人士，向长期关心支持我市建设的港澳台同胞、海外侨胞和国内外友人表示衷心的感谢和崇高的敬意！

　　我们也清醒地看到，我市经济社会发展还存在一些突出问题。主要是：经济结构调整和经济增长方式转变任务艰巨，自主创新能力亟待提升；城市承载能力和综合服务功能有待加强；城乡间、镇区间发展不平衡，农村工业化、城镇化和农业产业化水平亟待提高；资源和环境面临较大压力；社会转型时期的一些深层次矛盾逐步凸显；政府自身建设、社会管理和公共服务仍需进一步加强等。对此，我们要始终保持清醒的头脑，采取更加有力的措施认真加以解决。

今后五年的展望和2007年工作建议

　　今后五年，是我市全面实施和完成“十一五”发展规划，全面建设“两个适宜”城市、构建和谐中山的关键时期。新一届政府肩负着“实现新发展、开创新局面”的重任，必须切实增强忧患意识和发展意识，增强紧迫感和使命感，必须坚持以经济建设为中心，把构建社会主义和谐社会摆在更加突出的地位，奋发有为，开拓创新，以新的思路谋划发展，以新的举措建设和谐社会，实现经济社会新的发展和跨越。

　　今后五年政府工作的总体要求是：以邓小平理论和“三个代表”重要思想为指导，坚持以科学发展观统领经济社会发展全局，深入贯彻落实市第十二次党代会精神，以建设“两个适宜”城市，构建和谐中山，实现富裕安康为总目标、总任务，巩固经济社会协调发展成果，不断深化改革，提升对外开放水平；加快经济增长方式转变，增强自主创新能力，全面建设经济强市；统筹城乡、区域、人与自然协调发展，努力促进经济发展与社会事业全面进步，率先基本实现社会主义现代化。

　　未来五年政府工作的奋斗目标和主要任务是：

　　———增强综合实力，建设经济强市。全市生产总值年均增长10%左右，到2011年户籍人均生产总值超过11万元，初步建立“高、重、集、内”的新型产业体系；东部开发和带动作用成效明显；城市综合经济实力显著增强。

　　———完善城市功能，建设宜居城市。交通、水利、能源等功能性、枢纽性基础设施建设水平有较大程度的提高；城乡、区域更加协调发展，城市化水平达到78%以上；城市生态及环境指标处于较好水平，可持续发展能力不断增强。

　　———夯实科教基础，建设文化名城。推进经济社会发展的体制机制更加充满活力；以促进人的全面发展为核心的社会事业体系更加完善，进一步增强科技教育和人才实力，全面提高文化竞争力，促进市民的思想道德素质和科学文化素质明显提高。

　　———推进民主法治，建设和谐中山。政府管理更加科学化、民主化、法制化，市民更加充分和有序地参与城市发展及管理；社会保障网基本实现全社会覆盖，城镇登记失业率控制在3%以内；社会秩序和公共安全得到有效保障；全市人民始终保持昂扬向上的精神风貌。

　　———推动民生工程，实现富裕安康。城镇居民年人均可支配收入年均增长5%，农民人均年纯收入年均增长6%，广大市民普遍共享改革发展成果，普遍得到更多实惠，普遍过上更加富足的生活；形成较完善的卫生服务和医疗保健体系，健康城市建设卓有成效。

　　2007年是建设“两个适宜”城市，构建和谐中山的重要一年，做好今年的工作意义重大。2007年经济社会发展的主要预期目标是：全市生产总值增长 12%左右，工业总产值增长14%，全社会固定资产投资增长6%，外贸出口增长15%，万元生产总值能耗下降3.9%，城镇居民年人均可支配收入增长 5%，农民人均年纯收入增长6%，城镇登记失业率控制在3%以内，人口自然增长率控制在6.25‰以下。

　　2007年要在原来工作的基础上，承前启后，继往开来，努力实现新的发展。根据市第十二次党代会提出的总目标、总要求，我们要贯彻落实“科学发展、改善民生、促进和谐”的工作方针。在工作中要正确处理好“五个关系”：一是经济发展中“好”与“快”的关系，确保经济增长质量与效益的统一；二是发展动力中 “外”与“内”的关系，实现内外源经济的协调发展；三是城市建设中“建”与“管”的关系，把加强重大基础设施建设与科学有效的管理结合起来；四是社会管理中“宽”与“严”的关系，形成宽严有度、文明法治的管理秩序；五是利益协调中“收”与“放”的关系，既要加大对资源和财力的统筹力度，又要体现利益向下。要着力抓好以下工作：

　　一、加快经济结构优化升级，打造竞争优势明显的产业体系

　　全面推进产业高级化和适度重型化。要促进产业转型升级，打造产业发展新平台，才能有效增强城市综合竞争力。要坚持创新发展，切实转变增长方式。市财政安排6700万元用于技改贴息、扶持装备制造业和中小企业发展，推动工业向高集聚、高科技、高附加值转变。培育和发展健康医药产业，加快华南现代中医药城和中药现代化工程技术研究院建设步伐，今年在基础设施建设、项目投产、新药产业化方面开个好头，切实把中医药产业作为今年我市高新技术产业发展的重点，并在今后几年内成为我市具有发展潜力、特色明显、竞争力强的新增长点。加快建设装备制造工业研究院，争取在装备制造业、五大支柱产业以及光机电一体化、新材料、新能源、节能与环保等新兴产业的共性技术和关键领域取得突破，推动“中山制造”向“中山创造”转变。今明两年市财政每年安排不少于5500万元投入信息化建设，以信息化推动工业化，全面提升企业竞争力。积极实施技术标准战略，鼓励企业通过参与各级各类标准的制定占领行业制高点，加快建设国家级灯具产品、五金产品检验检测中心。继续实施名牌带动战略，提高品牌产品占有率和竞争力。推动装备制造业特别是精密装备制造业发展，提升装备制造业的研发、制造、系统集成和配套能力，建设好国家火炬计划中山（临海）装备制造业基地。大力发展高新技术产业，重点发展集成电路、通信、计算机和汽车电子产品，加快发展软件、动漫及数字视听、数字家电产业，2007年高新技术产品产值增长16%。全面提升工业园区和产业聚集区建设水平，增强承载大产业、大项目的能力。

　　继续培育壮大产业集群，进一步提升专业镇发展水平。专业镇是我市产业的一大特色，是凝聚我市工业竞争优势的最重要的载体。要围绕产业集群目标，把专业镇做大做强做精做专。引导各专业镇产业聚集区根据特色产业延伸产业链条，着力巩固薄弱环节和弥补断裂环节，形成完整的产业链和价值链。加快专业镇各类专业市场集群建设，为工业发展提供交易平台。扶持和吸引龙头企业，发挥带动和示范作用，鼓励中小企业联手打造区域品牌。积极引导西北部家电、五金、灯饰产业通过资源整合实现强强联合，加快创建全国集群创新试点。设立专业镇特色产业发展专项资金，加强专业镇科技创新平台建设，推动专业镇从简单的产业集聚向现代产业集群转变。加强专业镇共用基础设施及需求的研究和投入，完善技术研发、职业教育、信息服务、质量检测、物流配送、设计创意、金融法律等产业服务平台。

　　提升产业组织效率，促进现代服务业实现较快发展。坚持“二、三、一”产业发展方针，推动产业价值链从生产领域向服务领域延伸。以发展现代物流业、商贸服务业、旅游业为重点，以建设服务业集聚区为突破口，大力发展生产性和消费性服务业，进一步提升产业和城市的服务功能。加快东部、中部、西北部物流集聚区、物流市场和市保税物流中心建设，提升物流业信息化、标准化水平，继续推进“万村千乡市场工程”。优化商业网点布局，规划建设长堤路、富华道等商业圈，实施“振兴老字号工程”。推动区域旅游合作，充分利用外资、民资开发旅游资源。适度发展房地产业。积极培育会计、法律、科技中介、信息咨询、广告策划等中介服务业，引进一批境内外知名的中介机构，建设中介服务公共信息平台和商务集聚区，推动写字楼经济加快发展。壮大会展经济，继续办好3·28招商经贸洽谈会及休闲服装、灯饰、电子产品、食品等专业会展。优化消费市场环境，促进汽车、住房、康体、教育、文化、信息等消费增长，增强消费对经济的拉动作用。

　　突破资源、能源等要素瓶颈，大力发展循环经济。实施严格的土地管理制度，做到科学布局、集约利用，加大闲置土地处理力度，盘活存量土地，科学调控房地产土地供应，提高工业用地的投资强度和工业厂房的建筑容积率，确保土地供应向产业带动能力强、产出效益好、资源能源消耗少的大项目倾斜，提高土地产出率。切实提高对经济运行的监测分析和综合协调能力，加快电源电网及能源基础设施建设，确保电力和成品油稳定安全供应。大力发展“总部经济”，继续做好产业向市外及在镇区间的转移工作。完善能耗指标公布和考核制度，重点抓好工业、建筑、交通、商业、农村、政府机构等领域的节能工作，积极构建节能型产业体系，建设节约型社会。

　　二、扎实推进新农村建设，促进城乡、区域协调发展

　　构筑区域化布局、产业化经营和社会化服务的现代农业新格局。增强农业科技创新和转化能力，大力发展高产、优质、高效、生态、安全的现代农业。切实保护耕地特别是基本农田，确保完成省下达的粮食生产任务。加快农业产业化步伐，建设好规模化农业生产基地，培育更多农业龙头企业、农民专业合作经济组织和农业协会，设立发展农产品专业市场和物流业专项资金，鼓励镇区依托生产基地建成一批专业市场，建立和完善农业社会化服务体系。健全农产品质量检验检测体系，确保农产品质量安全。

　　推进农村基础设施建设，改善农村生产生活条件。加大政府投入，激活社会投入，引导农民自主投入，确保农村基础设施建设投入逐年稳步增长。继续推进“农村五项工程”，加强农村危桥（涵）改造和内河涌整治。推进“村改居”、城中村改造、中心村建设和农民集中居住小区建设，加快新农村建设示范点的规划和建设。加强镇村环境整治，争取用3年左右的时间使农村面貌有较大改观。

　　积极推进农村各项改革，完善农业农村投入和保护政策体系。继续坚持工业反哺农业、城市支持农村和多予少取放活的方针，把基础设施和社会事业发展的重点转向农村。鼓励社会各类资金投向农村，逐步建立多样化的农业农村投入新机制。深化农村股份合作制改革，探索股份合作组织向现代企业、社会经济组织法人过渡。完善农村社会治理结构，鼓励、引导有条件的镇区实行村级核算，推动社区经济事务与社会管理事务相分离。积极化解镇村集体债务，扶持经济欠发达村在两年内集体经济收入达到100万元以上。

　　培育新型农民，提升农村现代文明水平。深入推进“三个一”工程（每个农民都有一份工作，一份养老保险，一份医疗保险），努力实现农民老有所养、病有所医、劳有所作。加大农村劳动力职业技能培训力度，鼓励农民外出承包土地，支持农民自主创业。继续推进农村养老和医疗保险事业，建立覆盖全市、相互衔接、互为补充的农村社会保障体系。探索有效的农民教育方式，引导农民树立公民意识、勤勉意识、自立意识、合作意识、法治意识、责任意识。

　　三、加快建设重大功能性基础设施体系，提升城市现代化水平

　　着力建设对经济拉动力强的重大功能项目，完善城市基础设施体系。按照优化布局、配套成网、提升功能、适度超前的要求，促进基础设施承载能力的全面提升。改革路桥建设体制，成立投资主体明确、融资能力较强、责任职能清晰、资产管理科学的路桥集团公司，统筹推进全市路桥建设。完善交通设施体系，全面建设 “四高速五横六纵九加密”干线公路网，构建立体化、区域化、功能化的公路平台体系，努力实现与周边城市道路的“无缝”对接。确保古神公路年内全面通车，完成南北外环线建设，全面启动新沙水公路、福源路建设和岐江公路改造等工程，配合省建设好广珠城际轨道交通、太澳高速公路（中山段）等项目。建设总面积达4 万平方米以上的若干个大型停车场，解决中心城区“停车难”问题。进一步完善城区交通组织和交通形态，继续推进道路畅通工程，标本兼治，有效解决“行车难” 问题。坚持“公交优先”发展，深化公交体制改革，实施“公交一盘棋”，优化线网布局，提高公交智能化水平。加强城市防灾减灾体系建设，完善各种应急预案，增强抵御各种自然灾害的能力。确保各级财力投入，市财政累计投资20亿元按照“50年一遇”标准建设中顺大围达标加固及西河水闸重建等水利工程，进一步增强防洪和水资源安全保障能力。完善能源设施体系，加快嘉明电厂扩建、市域天然气利用工程、500千伏桂山（三乡）变电站及德隆、宝山、菊城等三个220千伏输变电工程新建和中山站改造工程。完善民生设施体系，加快博览中心、市一中初中部建设，启动纪中、二中扩建及拆迁改造，从2007年起用三年时间完成中心城区预制构件公房改造，完成新颐老院建设和东门、南门、泰安市场改造。继续推进“供水一盘棋”，加快实现全市安全优质供水。年内实现“村村通自来水”。

　　以东部开发带动组团发展。加快四大组团特别是东部组团的战略研究和各专项规划编制工作，明确各组团的战略措施、战略重点，优化组团产业布局，统筹组团内部基础设施建设和管理，形成优势互补、资源共享、产业联动、相互促进的发展格局。充分发挥东部组团在我市产业升级中的示范带动作用，全面推进“一港一路八园”建设，构建有利于使东部组团形成以健康医药产业基地、绿色能源基地、石化工业基地、装备制造工业基地为载体的高新技术产业带，重点发展电子信息、生物医药、现代包装印刷、精细化工材料、汽车配件、新能源新材料等产业的较完备的基础设施。充分发掘岸线和港口资源，推进中山港二期扩建工程，进一步提高港口吞吐能力。优先推进镇际基础设施建设，强化各组团对生产要素的承载功能，加快西北部、中部、南部组团发展。

　　营造人与自然和谐的城市环境。积极创建全国生态市，坚持从源头上防治污染和保护生态，构筑良性循环的城市生态系统。继续加强以岐江河综合整治为重点的水环境综合整治，采取最严格的措施保护饮用水源，打造“绿色”健康河流。继续大力创建全国环境优美乡镇，努力改善农村生活环境。实施污染物排放总量控制，积极筹建工业污染源、汽车尾气、河流水质、空气质量等在线监测系统，提高突发环境污染事故预警和应急处置能力。加强五桂山自然生态保护区管理，推进第二重山森林改造、湿地保护和红树林建设。重点推进北部、南部组团垃圾综合处理基地、珍家山污水处理厂一期等重大环保工程。全面开征污水处理费，全面加快镇区污水处理厂建设，限期完成省下达的污水处理任务。继续推进“绿化中山大地工程”，加快文笔山大风车公园、古香林郊野公园、大尖山公园、马山公园、五马峰公园建设，使市中心城区绿化覆盖率提高到39%，人均公共绿地达10.8平方米。

　　切实提高城市经营和管理水平。深化投融资体制改革，大力推行城市公共设施政府项目代建制、BT、BOT、TOT及直接融资等多种建设模式，引导商业银行、商业保险资金参与基础设施建设，多渠道解决好城市建设资金问题。加强城市规划对城市管理的调控作用。坚持建管并重，切实加强城市管理，促使城市交通畅顺通达。强化土地收购储备制度，年内实现新增土地储备1万亩。加强城市风貌建设，切实保护好历史文化遗产和古建筑、古文物。加强图书馆、科学馆、体育馆、文化艺术中心等场馆的科学管理和运营。积极推动城管重心下移，完善以镇、村为管理主体，多部门综合治理的城市管理联动机制，提高城市管理综合执法水平。

　　四、坚持“内外并重”，建立更具活力更加开放的体制机制

　　加快转变外贸增长方式，提升招商引资水平。积极参与“泛珠三角”以及中国———东盟自由贸易区等经济合作，提高区域合作层次和水平。优化利用外资结构，力争实际吸收外商直接投资7亿美元以上。继续推动从招商引资到招商选资，从“以地招商”到“节地招商”，从项目招商到产业招商的转变。坚持“引资”与 “引智”相结合，鼓励外商在我市设立研发中心、地区总部。健全外贸促进服务体系和公平贸易支持体系，支持企业组建行业协会和商会，积极应对贸易壁垒和国际市场波动。优化进口结构，大力引进先进技术、关键装备和重要原材料。加强对外资企业的管理，促使其规范、有序发展。继续加强“电子口岸”建设，提升“大通关”水平。

　　积极推动本土经济发展壮大。继续深化公有资产管理体制改革，健全监督体制，确保公有资产保值增值，公有资产要加快退出一般竞争性领域。鼓励民间投资进入基础设施、公用事业等领域，引导民营经济进入先进制造业、高新技术产业和现代服务业。积极引入大型国有企业，形成更加紧密的战略合作伙伴关系，带动本土经济的优化升级。大力发展资本市场，激励优质企业上市，加速中小企业发展壮大。积极发展“担保经济”。积极引进国内股份制银行和外资银行，促进金融产品、金融服务多元化，拓宽民营企业融资渠道。推动民营企业通过产业配套、战略合作和投资参股等形式，实现与外资产业链的联接。加强引导和培训，提升民营企业家的整体素质和实力。

　　加快形成有利于转变经济增长方式、激发全社会创造力的体制机制。继续深化行政管理体制改革，促进政府职能转变，进一步加强对规划和资源的调控力度，完善市镇两级利益机制。深化科技、文化、教育、医疗卫生管理体制改革，积极稳妥地分类推进事业单位改革。完善公共财政体制，健全激励型转移支付制度，开展市级财政对镇区实行国库集中支付改革试点，财政支出更多向“三农”、科教文卫、社会保障、公共管理、公共基础设施等领域倾斜。加强税收征管，规范非税收入收支管理。继续整顿和规范市场经济秩序，健全市场监管体制。加快建设社会信用体系。

　　五、加强创新能力建设，推动社会协调、可持续发展

　　增强科教实力，建设创新型中山。强化企业的自主创新主体地位，支持大中型企业组建工程中心、博士后工作站等研发机构。加强科技创新基地和共性技术平台建设，健全面向企业特别是中小企业的技术咨询、技术转让等社会服务体系。逐步构建完善的成果转化体系，加快建设特色产业科技创业园、高新技术企业孵化器等成果中试基地。安排科技经费1.9亿元，强化知识产权保护，积极发展创新风险投资。倡导创新精神，培育创新文化，营造有利于创新创业的良好环境。加快建设 “人才强市”，积极引进和培育高科技、高层次和产业发展急需的各类人才。

　　加快推进教育现代化进程。坚持教育优先，促进教育公平，实现各级各类教育全面协调发展，安排6100多万元用于落实好免费义务教育。继续实施“振兴初中行动计划”，推进示范性高中建设。深入推进“三名”工程（名校、名师、名校长），建设高素质的教师和校长队伍。继续扩大高等教育规模，支持广东药学院中山校区二、三期建设和电子科技大学中山学院扩建，努力形成与创新型城市相适应的高等教育体系。超常规发展职业技术教育，组建职业技术教育集团，建设市级实训中心，加快中山职业技术学院三期建设，使中高职教育在校学生人数达4万人以上。规范发展各类非普通教育，形成专业化、社会化和市场化的成人教育机制，建设终身教育体系和学习型城市。

　　大力发展文化事业和文化产业。努力提升城市文化内涵和文化品位，提高广大市民文化素养，重点建设和启动博物馆群、档案馆等公益性文化工程。积极开展历史文化资源的开发利用，深入推进香山文化的传承和发展，加快申报“中国历史文化名镇（村）”工作。启动文化产业园建设，推动文化产业和创意经济发展。培育面向市场的竞争主体，大力发展新闻出版、广播影视、文化娱乐、文化旅游、文化会展等产业。全面推进有线数字电视整体转换。

　　促进各项社会事业协调发展。继续加强城乡体育设施建设，深入推进全民健身运动，提高竞技体育水平。加强计划生育管理，实施利益导向型人口控制计划，稳定人口低生育水平，继续推进出生缺陷干预工程，努力提高人口素质。加强国防动员基础设施建设和国防教育，巩固双拥工作成果。切实保障妇女、未成年人合法权益。做好老龄、残疾人、民族宗教、外事、侨务、台务、老干、统计、气象、人防、档案、地方志、文史等工作。

六、让人民群众共享改革发展成果，促进社会公平

　　完善分配制度，提供更多就业机会。着力提高低收入者收入水平，逐步扩大中等收入者比重。推进公务员工资制度改革并建立工资正常增长机制，改革和完善事业单位工作人员收入分配制度。大力开发公益性就业岗位，积极发展社区就业。安排1800万元作为就业再就业专项资金，重点帮助“零就业家庭”、农村贫困家庭、“4050”人员等困难群体就业。实施城乡青年技能培训、创业富民工程和技能人才振兴计划，全年培训城乡劳动者3万人次，扶持贫困家庭子女免费就读市高级技工学校。鼓励企业吸纳本市城乡劳动者就业。完善公共就业服务体系。做好城乡退役士兵免费职业技能培训工作。

　　加快建立健全覆盖全市的社会保障体系和公共救助服务体系。逐步建立社会保险、社会救助、社会福利、慈善事业相衔接的覆盖城乡的社会保障体系，实现社会保障“全覆盖”。年内安排1.74亿元专项用于社会保障工作，不断完善养老、医疗、失业、工伤、生育等保险制度，提升基金保障水平和抗风险能力，扩大社保覆盖面，实现社会保险参保达380万人次以上。加快推进农村基本养老保险工作，力争参保农民达38万人以上，逐步推进城镇与农村社会保险并轨。加快推进 “金保工程”。健全城乡统筹的社会救助体系，安排2600多万元用于城乡社会救济和最低生活保障，继续推进建设“双困户”住房、扶困助学、救助困难群体等工作，完成农村低保危房改造。

　　加快医疗卫生事业发展，着力解决“看病贵”问题。大力提高城乡居民医疗健康水平，增强突发公共卫生事件应急、疾病预防控制和医疗救治服务能力。着力提高农村公共卫生水平，推进镇村医疗卫生管理改革，完善社区卫生服务功能。年内完成第二人民医院建设，加快推进新中医院、人民医院和博爱医院（扩建）等重点医疗卫生工程。巩固和发展新型农村合作医疗制度。继续推进城乡居民住院基本医疗保险，力争2007年底前参保人数达35万。继续推行济困助医及廉价医疗服务，完善有利于广大市民及时就医、安全用药、合理负担的医疗卫生制度。积极发展中医药事业。巩固创建卫生城镇成果，继续大力开展爱国卫生运动。

　　七、营造良好发展环境，全面建设“平安家园”

　　提升全体市民道德素质，大力倡导新时期中山人精神。继续深入开展“爱国、守法、诚信、知礼”现代公民教育活动，弘扬符合传统美德和时代精神的道德规范和行为规范，引导广大干部群众知荣辱、树新风、求和谐、促稳定。注重人文关怀、心理疏导，倡导奉献意识、集体意识和责任意识，塑造自尊自信、理性平和、健康向上的社会心态。大力普及科学知识、宣传科学精神，促进全社会形成健康文明的生活方式。高度重视未成年人思想道德建设，加强综合活动场所管理。

　　发挥社区和各种社会组织作用，妥善处理社会矛盾。深入开展平安和谐社区创建活动，把社区建设成为管理有序、服务完善、文明祥和的社会生活共同体。积极培育和规范管理各类社会组织，充分发挥社团、行业组织和社会中介组织在和谐社会建设中的作用。深入开展城乡志愿服务活动，建立与政府服务、市场服务相衔接的志愿服务体系。继续完善四级信访网络，努力畅通群众诉求渠道，完善群体性事件预警处置机制，有效预防化解各类矛盾。做好人民调解工作，完善劳动仲裁制度，建立新型劳动关系调整机制。加强征地管理，严格落实“三条红线”，避免征地拆迁问题造成新的社会矛盾。

　　加强社会治安综合治理，切实解决好群众反映强烈的治安问题。健全一把手负总责的“大综治”机制，提高依法管理社会的能力和水平。大力推进“平安中山” 建设，重拳打击各种违法犯罪，增强人民群众的安全感。加强对治安重点场所、出租屋、特种行业的管理，加快推进“五张网络”建设，完善覆盖城乡的社会治安防控网络系统。实施“科技强警”，年内建成3000个以上社会治安视频监控点，尽快建成完善高效的社会治安视频监控和快速反应系统。强化基层基础工作，充实一线警务力量。力争破案绝对数增幅在5%以上，命案破案率达89%以上，有效维护公共安全。

　　继续以铁的手腕抓好安全生产，健全公共安全防控体系。始终坚持“安全生产责任重于泰山”的观念，进一步健全各级安全生产责任体系、重大危险源监管体系及重大事故预防控制体系、安全生产事故灾难应急救援体系，确保全市生产总值安全事故死亡率同比下降10%以上。全面强化食品药品安全管理，做好重大动物疫病防控工作。严格安全执法，深入开展重点行业和重点单位安全隐患专项整治，加大交通、消防安全监管力度，遏制重特大安全事故的发生。

　　八、打造“阳光政府”，着力提供高效优质的公共产品和服务

　　加强政府自身建设，建设学习型、创新型、服务型政府机关。在政府工作中全面推行“目标明确、责任清晰、制度保障、绩效考核”的目标管理。使政府的每一项工作有目标要求，有责任主体，有工作进度，有监督考核，有奖惩机制，有责任追究，实现政府行政管理的精细化、流程化、标准化、协同化。推行行政问责制，健全行政责任体系。全面落实重大建设项目责任制和政府投资项目责任制，加强绩效考核和行政效能监察，鼓励行政部门进行工作创新。加强行政监督监察和责任追究，进一步推动电子政务，打造“阳光政府”。

　　深入推进依法治市，把依法行政贯穿于政府工作的各个方面、各个环节。自觉接受人民代表大会及其常务委员会的法律监督，人民政协和各民主党派的民主监督以及司法监督、群众监督、舆论监督。深入贯彻实施国务院《全面推进依法行政实施纲要》，规范行政许可，建立重大决策、重大工程建设项目社会稳定风险评估制度。完善行政执法责任制，加强行政复议和行政应诉工作，做到严格执法、公正执法、文明执法。继续做好督查、督办和群众投诉调处工作。

　　加强民主政治建设，提高政务公开水平。继续规范和完善重大决策出台的程序和规则，建立完善重大问题立项调研、专家咨询、社会公示和听证制度，切实做到科学行政和民主行政。积极畅通依法有序的民意表达渠道，坚持市政府领导牵头办理意见和议案提案制度。扩大人民群众在法律范围内有序的政治参与，充分发挥政府网站、《政报》、政府新闻发布、城市论坛等公开渠道的作用，加强与群众互动交流。进一步增强群众观念，深入基层，深入群众，倾听群众呼声，关心群众疾苦，完善市长接待日和市长下访日制度。

　　建设清正廉洁、干净干事的公务员队伍。完善政府采购有形市场、建设工程招投标、经营性土地使用权出让等制度。深入贯彻《公务员法》，切实把机关作风建设作为一项常抓不懈的工作，健全作风建设的领导机制和工作机制，大力倡导勤奋好学、学以致用，真抓实干、务求实效，顾全大局、令行禁止，秉公用权、廉洁从政，心系群众、服务人民，艰苦奋斗、勤俭节约，发扬民主、团结共事，生活正派、情趣健康的良好风气。要进一步加强廉政建设和反腐败斗争，坚决纠正损害群众利益的不正之风，严肃查处违反行政纪律和侵害人民群众利益的案件，在建设责任政府、高效政府、法治政府、廉洁政府上迈出更大步伐。

　　2007年是本届政府的开局之年，市政府把今年确定为“工作落实年”，要总体规划、全面协调、加快推进市委、市政府十项重要民生工程的启动和实施，一是网络化交通基础设施工程；二是“平安中山”工程；三是社会保障“全覆盖”工程；四是全民健康工程；五是全民宜居工程；六是“绿色中山”工程；七是职业技术教育“超常规发展”工程；八是城市文化工程；九是城乡水利防灾减灾工程；十是旧城改造工程。

　　各位代表，中山未来五年发展的蓝图已经绘就。新一届政府面对新的挑战，肩负神圣使命，我们一定要站在新起点上继往开来，务实拼搏，把全部心思倾注到干事业上，把全部精力集中到办实事上，把功夫真正下到抓落实上，在真抓实干中开拓前进！让我们紧密团结在以胡锦涛同志为总书记的党中央周围，高举邓小平理论和“三个代表”重要思想伟大旗帜，在省委、省政府和市委的坚强领导下，团结带领全市人民，全面落实科学发展观，振奋精神，扎实工作，为建设“两个适宜”的和谐中山，为建设富裕安康的美好家园而努力奋斗！
